# Comparison of breast cancer patients who underwent partial mastectomy (PM) with mini latissimus dorsi flap (MLDF) and subcutaneous mastectomy with implant (M + I) regarding quality of life (QOL), cosmetic outcome and survival rates

**DOI:** 10.1186/s12957-020-01858-z

**Published:** 2020-05-05

**Authors:** Vahit Ozmen, Serkan Ilgun, Burcu Celet Ozden, Alper Ozturk, Fatma Aktepe, Filiz Agacayak, Filiz Elbuken, Gul Alco, Cetin Ordu, Zeynep Erdogan Iyigun, Hocaoglu Emre, Kezban Pilancı, Gursel Soybir, Tolga Ozmen

**Affiliations:** 1 Department of Surgery, Istanbul Faculty of Medicine, Istanbul University, Istanbul, Turkey; 2 Istanbul, Turkey; 3 Department of General Surgery, School of Medicine, Demiroglu Bilim University, Istanbul, Turkey; 4 Department of Plastic and Reconstructive Surgery, School of Medicine, Altinbas University, Istanbul, Turkey; 5 Department of General Surgery, School of Medicine, Biruni University, Istanbul, Turkey; 6 Department of Pathology, Gayrettepe Florence Nightingale Hospital, Istanbul, Turkey; 7 Department of Radiology, Istanbul Florence Nightingale Hospital, Istanbul, Turkey; 8 Department of Radiology, Gayrettepe Florence Nightingale Hospital, Istanbul, Turkey; 9 Department of Radiation Oncology, Gayrettepe Florence Nightingale Hospital, Istanbul, Turkey; 10 Department of Medical Oncology, Gayrettepe Florence Nightingale Hospital, Istanbul, Turkey; 11 Department of Physical Therapy and Rehabilitation, Istanbul Florence Nightingale Hospital, Istanbul, Turkey; 12 Department of Plastic & Reconstructive Surgery, Istanbul Florence Nightingale Hospital, Istanbul, Turkey; 13 Department of Medical Oncology, Bahcesehir Memorial Hospital, Istanbul, Turkey; 14 Department of General Surgery, Sisli Memorial Hospital, Istanbul, Turkey; 15 Department of Surgery, Miller School of Medicine, University of Miami, Miami, Florida, USA

**Keywords:** Mini latissimus dorsi flap, Breast-conserving surgery, Subcutaneous mastectomy, Implant reconstruction; quality of life, EORTC-QLO C30, EORTC-QLO BR23, Cosmetic evaluation, Japanese breast cancer society cosmetic evaluation scale

## Abstract

**Purpose:**

The latissimus dorsi muscle has long been used in breast cancer (BC) patients for reconstruction. This study aimed to compare early stage BC patients who had partial mastectomy (PM) with mini latissimus dorsi flap (MLDF) and subcutaneous mastectomy with implant (MI) with respect to quality of life (QoL), cosmetic outcome (CO), and survival rates.

**Patients and methods:**

The data of patients who underwent PM + MLDF (Group 1) and M + I (Group 2) between January 2010 and January 2018 were evaluated. Both groups were compared in terms of demographics, clinical and pathological characteristics, surgical morbidity, survival, quality of life, and cosmetic results. The EORTC-QLQ C30 and EORTC-QLO BR23 questionnaires and the Japanese Breast Cancer Society (JBCS) Cosmetic Evaluation Scale were used to assess the quality of life and the cosmetic outcome, respectively.

**Results:**

A total of 317 patients were included in the study, 242 (76.3%) of them in group 1 and 75 (23.6%) of them in group 2. Median follow-up time was 56 (14–116) months. There were no differences identified between the groups in terms of tumor histology, hormonal receptors and HER-2 positivity, surgical morbidity, and 5-year overall and disease-free survival. Group 2 patients were significantly younger than group 1 (*p* = 0.003). The multifocality/multicentricity rate was higher in group 2 (*p* ≤ 0.001), whereas tumor size (*p* = 0.009), body mass index (BMI, *p* = 0.006), histological grade (*p* ≤ 0.001), lymph node positivity (*p* = 0.002), axillary lymph node dissection (ALND) rate (*p* = 0.005), and presence of lympho-vascular invasion (LVI, *p* = 0.013) were significantly higher in group 1. When the quality of life was assessed by using the EORTC QLQ C30 and BR23 questionnaires, it was seen that the body image perception (*p* < 0.001) and nausea/vomiting score (*p* = 0.024) were significantly better in PM + MLDF group whereas physical function score was significantly better in M + I group (*p* = 0.012). When both groups were examined in terms of cosmesis with JBCS Cosmetic Evaluation Scale, good cosmetic evaluation score was significantly higher in patients in MLDF group (*p* = 0.01).

**Discussion:**

The results of this study indicate that in comparison to M + I procedure, the PM + MLDF procedure provides significantly superior results in terms of body image and cosmetic result with similar morbidity and oncologic outcomes. In selected patients with small breasts and a high tumor/breast ratio, PM + MLDF may be an alternative to subcutaneous mastectomy and implant.

## Introduction

Breast cancer (BC) is the most common cancer and cause for cancer-related death among women worldwide [1, 2]. Surgical treatment of BC has evolved from radical mastectomy to breast-conserving surgery (BCS) in the last four decades. On the other hand, new effective chemotherapeutic drugs have prolonged the life expectancy of patients and reduced locoregional recurrence rates [3]. Along with these improvements, cosmetic outcomes and oncoplastic breast surgery (OBS) have gained more importance [4]. The primary aim of OBS is to obtain excellent cosmetic results together with acceptable surgical outcomes [5, 6]. To be able to ensure a good cosmetic result, intra-glandular or autogenous muscle flaps have been used to replace volume loss following wide excision [6, 7]. Small volume losses where the breast tumor is small can be successfully repaired using intra-glandular flaps (volume replacement). However, for large volume losses especially in patients with small breast and large tumor, autogenous muscle flaps may be used to fill the tumor cavity (volume displacement).

The first literature report of the use of latissimus dorsi muscle skin flap was the closure of a mastectomy defect by Iginio Tansini in 1906 [8]. In the 1980’s, the latissimus dorsi myocutaneous flaps became the preferred autogenous reconstruction method for breast reconstruction after mastectomies, and it started to be used also in partial mastectomies after 1990’s [9–12]. Volume replacement with MLDF provides a cosmetically successful breast reconstruction especially in patients with large tumor/breast volume ratio, who otherwise would be referred to mastectomy [13–16].

The possibility to fill the tumor cavity by using autologous flaps in selected patients increases the breast conservation ratio and enables this intervention to be a serious alternative to subcutaneous mastectomy and implant procedures [13–18]. Subcutaneous mastectomy with latissimus dorsi muscle flap usually requires implant and adds additional cost. It also has many other morbidities if we compare with PM + MLDF such as skin flap and nipple-areola necrosis, increase in duration of surgery, hospitalization, and recovery period to start systemic treatment. Saving nipple-areola complex together with most of the breast tissue has also positive psychologic effect on patients’ mood. Subcutaneous mastectomy and implant usually causes asymmetries and requires additional cosmetic approaches in contralateral breast for better and symmetric cosmetic outcomes. Most of patients with subcutaneous mastectomy and implants always feel a foreign body instead of their own tissue for the rest of their lives. Breast conserving surgery with PM + MLDF also saves body image with autolog graft and keeps oncological principles. However, no studies in the literature compared these two procedures. This study aimed to compare the patients with PM + MLDF with the patients with M + I in terms of surgical morbidity, quality of life (QoL), cosmetic appearance, and recurrence and survival rates and to give a message to breast surgeons that the breast may be preserved in patients who do not have a chance for volume displacement to fill the tumor cavity and require mastectomy and implant.

## Patients and methods

This is a retrospective review of early BC patients (cStage I, IIA) operated at our institution between January, 2010 and January, 2018. The patients were divided into two groups according to the surgical procedure performed: group 1, PM + MLDF; group 2, M + I. Demographic, clinical, and pathological characteristics of patients; surgical morbidities; locoregional recurrence; and survival data were compared. The patients were evaluated in tumor board preoperatively, and it was decided that PM + MLDF may be an alternative to M + I for selected patients. All PM + MLDF and subcutaneous mastectomies were done by a single surgeon (VO) who have more than 30 years of experience in breast surgery. Plastic surgeon was involved in surgery to insert implant after subcutaneous mastectomy, and all reconstruction with implant procedures were performed by the same plastic surgeon (BCO). The breast and tumor volumes were calculated by using a software provided by Varian Inc.® and a specific volume formula (V = 4/3 πr3), respectively.

Both two procedures were explained to patients, and their choices were recorded. They signed informed consent form. Ethical committee approval was obtained.

Contraindications for PM + MLDF were diffuse microcalcifications and extensive multicentric cancer requiring mastectomy, patients’ desire, locally advanced BC, or inflammatory BC. Last two contraindications were also valid for M + I. There was no bilateral BC in the two groups.

The EORTC QLQ-C30 [19] and EORTC QLQ-BR23 [20] questionnaires and JBCS Cosmetic Evaluation Scale [21] were used to asses QoL and cosmetic outcome, respectively. The quality of life questionnaire was conducted by an independent expert nurse. The QLQ-C30 included 30 questions. These questions were comprised of functional scale questions (physical function, role function, emotional function, cognitive function, and social function), three symptom scale questions (fatigue, nausea, vomiting, and pain) and six individual evaluation questions (dyspnea, insomnia, loss of appetite, constipation, diarrhea, and financial difficulties). The QLQ-BR23 questionnaire included 23 questions. These questions contained sub-sets including the functional scale (body image, sexual function, sexual pleasure, and expectations for the future) symptom scale, side effects due to systemic treatment, complaints regarding breast, complaints regarding the arm, and loss of hair. The quality of life score was calculated using values between 0–100 according to the calculation guidelines published in accordance with the QLQ-C30-23 [22]. The higher scores reflected a better function and quality of life.

The cosmetic evaluation was conducted by a plastic surgeon who was not part of the surgical team. All the cosmetic evaluations and quality of life evaluation were completed at least 1 year after the surgery.

### Peri-operative histopathologic evaluation

The surgical margins and sentinel lymph node(s) were evaluated intra-operatively by an experienced breast pathologist (FA). Re-excision was performed in the presence of a positive surgical margin. Negative surgical margin width was accepted as ≥ 2 mm and no tumor on ink before and after SSO and ASTRO consensus guideline [23, 24]. Subareolar margin was evaluated after subcutaneous mastectomy, and nipple-areola complex was excised if there was margin positivity. Blue dye, radioisotope, or both of them were used to find sentinel lymph node(s), and axillary clearance was performed in the presence of > 2 positive lymph node(s) after ACOSOG Z011 trial [25].

### Surgical technique

**Fig. 1:**
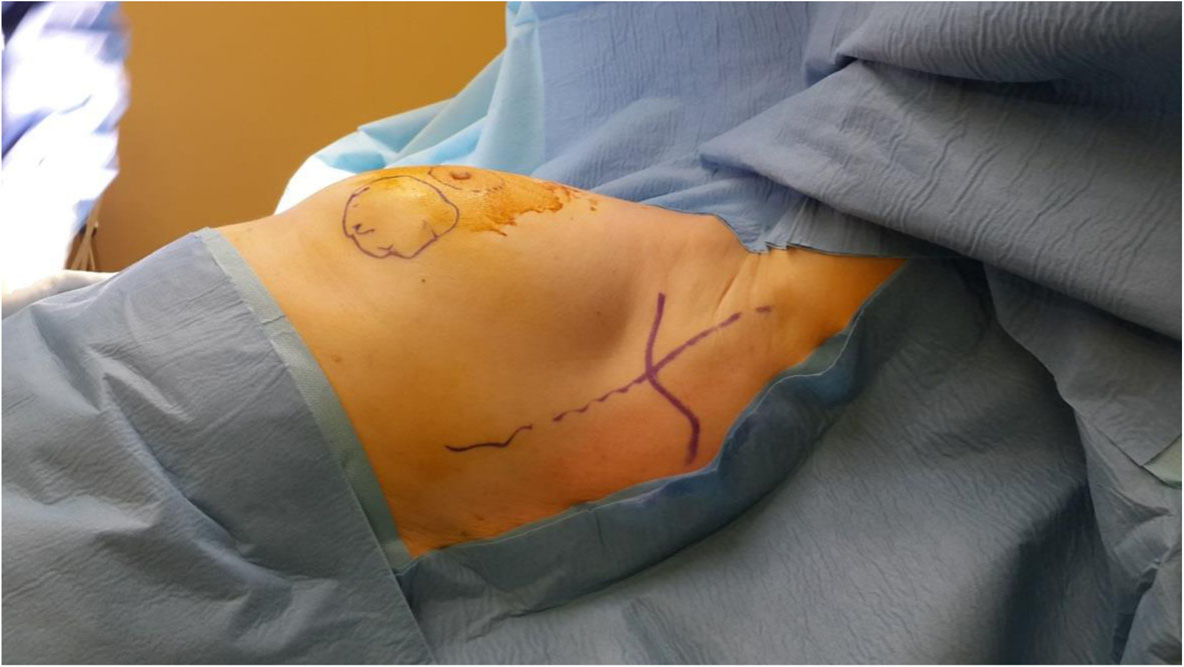
Position of a BC patient for PM and MLDF, a pillow is inserted behind the left scapula, and left fore-arm fixed the bar. Tumor is localized lower outer quadrant of the breast

**Fig. 2:**
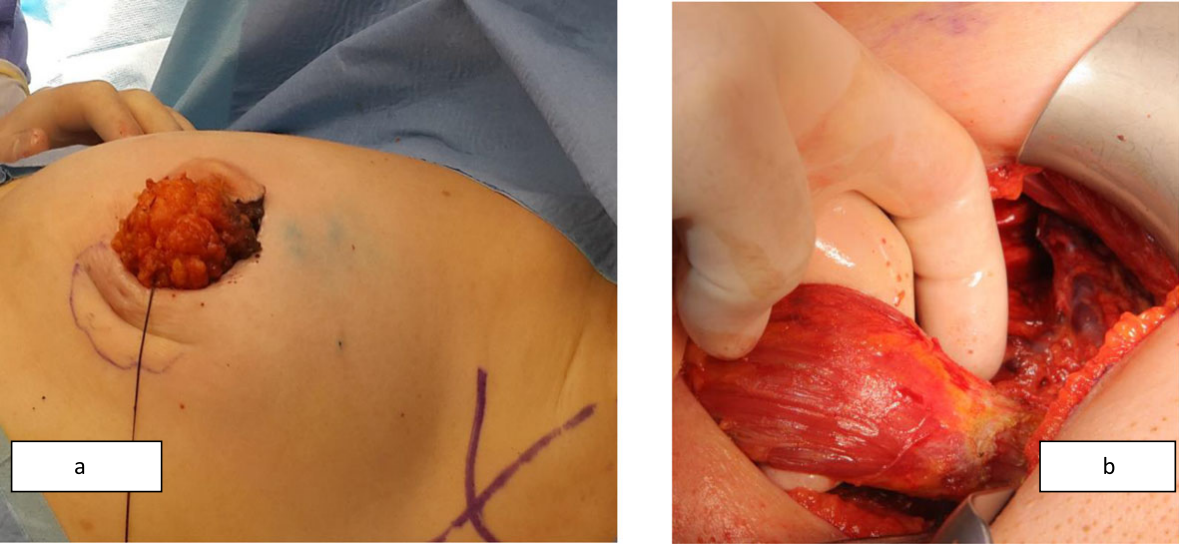
**a** The tumor is excised from circumareolar incision and **b** superior part of the muscle is identified and divided at its insertion to the humerus, and dissection is continued deep to reach the scapula **b**

**Fig. 3:**
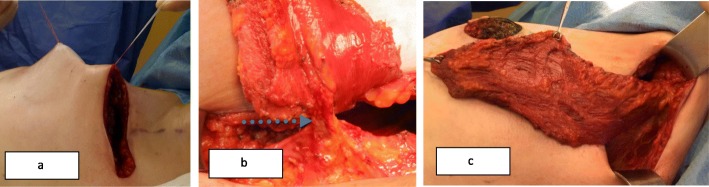
**a** A tunnel is created between tumor cavity and the axilla, **b** the arrow shows neurovascular pedicle of the muscle, and **c** mini latissimus dorsi flap is prepared and ready to fill the cavity **c**

**Fig. 4:**
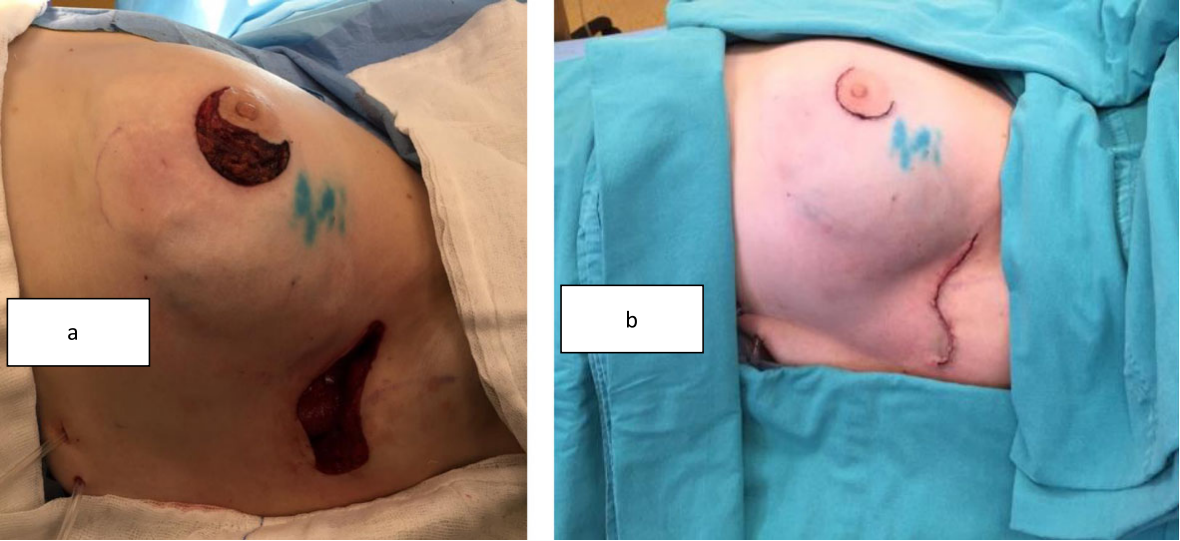
**a** The flap is inserted in the tumor cavity, fixed to the edge of pectoralis major muscle, and **b** Jackson Prett inserted and incisions are closed

**Fig. 5:**
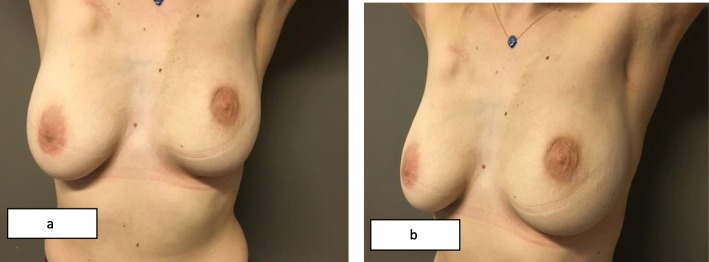
**a** Anterior and **b** lateral view of the same patient 1 year after the surgery

These two different surgical techniques were one-step procedures. PM + MLDF was given as an alternative to patients who were candidates for M + I due to diffuse multicentric disease and/or microcalcifications, high tumor/breast ratios (large tumor and small size breast), and patients’ desire to save their breasts, if it is possible. The surgical technique of PM + MLDF was explained in our previous study [15]. A position is given; tumor and sentinel lymph node (SLN) are excised from circumareolar and axillary incision and sent to intra-operative pathologic evaluation (Fig. 1 and 2a). After surgical margins and SLN evaluation, specimen is weighted. Superior part of the muscle is identified and divided at its insertion to the humerus, and dissection is continued deep to reach the scapula (Fig. 2b). A tunnel is created between tumor cavity and the axilla; neuro-vascular pedicle of the muscle is preserved; and mini latissimus dorsi flap is prepared and ready to fill the cavity (Fig. 3). The flap is inserted in the tumor cavity and fixed to the edge of pectoralis major muscle (Fig. 4a). Jackson Prett drain is inserted and incisions are closed (Fig. 4b). Anterior and semi-lateral view of the same patient one year after surgery is shown in Fig. 5.

Since it gives an opportunity to evaluate the axilla, subcutaneous mastectomy was mostly performed via lateral radial incision. Implant was inserted between pectoral muscles, and acellular dermal matrix was utilized in 17 (22.6%) patients. Nipple-areola complex reconstruction was performed after all treatments were completed.

### Radiation therapy

All patients in PM + MLDF group received whole breast and boost radiation therapy. Radiation to regional lymphatics was given to 23 (36%) patients in M + I group.

### Follow-up

The patients in this study were regularly followed by a specialized breast surgeon. Wound infection and dehiscence, flap necrosis, hematoma, and seroma were considered as signs of morbidity (complications in the early period). The drainage tubes were removed when the amount of seroma was less than 25 cc per day. The adjuvant treatment decision was made during the tumor board.

### Statistical analysis

SPSS version 22 (IBM Corporation) was used for statistical analysis. The variables were investigated visual (histograms, probability plots) and analytical methods (Kolmogorov-Simirnov/Shapiro-Wilk’s test) to determine whether or not they are normally disturbed. Descriptive analyses were presented using medians and interquartile range for non-normally disturbed and ordinal variables. Non-parametric data were evaluated using the Mann-Whitney *U* tests. The categorical variables were analyzed using the Chi-square test. The effects of surgical procedures on survival was investigated by using the log rank test. The Kaplan-Meier survival estimates were calculated. A *p* value of < 0.05 was considered statistically significant.

## Results

A total of 317 patients were included in the study with 242 (76.3%) of them in group 1 (PM + MLDF) and 75 (23.6%) in group 2 (M + I). Median follow-up time was 56 (14-116) months. The patient and tumor characteristics in two groups are shown in Table 1. There were no differences identified between the groups in terms of tumor histology, ER and PR positivity, and poor molecular subtype rates, but the number of patients with luminal A molecular subtype were significantly higher in M + I group (*p* = 0.013). The mean breast volumes were 537.22 ± 235.52 ml in group 1 and 613.75 ± 253.83 ml in group 2, respectively (*p* = 0.253). If there was only one tumor in the breast, the median excised tumor volumes with 10 mm safe margin in two groups were 41.6 ml in group 1 and 33.4 ml in Group 2 (*p* = 0.09), respectively. The tumor/breast ratio was higher in group 1 (0.077) than group 2 (0.054), respectively. The multifocal/multicentric cancer rates were very high in both groups, and there was significant difference between the two groups (25.9% vs 50%, *p* = 0.000). The patients in group 2 were significantly younger than group 1 patients. Body mass index (BMI, *p* = 0.006), advanced histological grade (*p* ≤ 0.001), lymph node positivity (*p* = 0.002), ALND rate (*p* = 0.005), and presence of lympho-vascular invasion (*p* = 0.013) were significantly higher in group 1 (Table 1).

**Table 1:** Comparison of patient and tumor characteristics in two groups

**Characteristics**		**Group 1**	**Group 2**	***p*value**
		**(PM + MLDF)**	**(M + I)**	
		(*n* = 242)	(*n* = 75)	
**Median age (year)**		45 (26–73)	42 (24–78)	**0.003**^a^
**BMI (kg/m2)**		24.4 (16.4–44.4)	22.5 (16.9–32.4)	**0.006**^a^
**Menopausal status**				
**Pre-menopausal**		166 (68.6%)	65 (86.7%)	**0.002**^**#**^
**Post-menopausal**		76 (31.4%)	10 (13.3%)	
**Tumor size (mm)**		23 (1–90)	20 (1–80)	**0.009**^a^
**Breast volume (ml)**		537.22 ± 235.52	613.75 ± 253.83	***p*= 0.253**
**Tumor focality**				
**Unifocal**		177 (74.1%)	37 (50%)	**0.000**^b^
**Multifocality/multicentricity**		62 (25.9%)	37 (50%)	
**Tumor Histology**				
**IDC**		204 (84.3%)	54 (72%)	0.056^b^
**ILC**		19 (7.9%)	10 (13.3%)	
**Others**		19 (7.9%)	11 (14.7%)	
**Histologic Grade**				
**HG I**		12 (5%)	7 (9.5%)	**0.000**^b^
**HG II**		92 (38.3%) ^a^	46 (62.2%) ^b^	
**HG III**		136 (56.7%) ^a^	21 (28.4%) ^b^	
**pT Stage**				
**T1**		106 (43.8%)	44 (58.7%)	0.052^b^
**T2**		126 (52.1%)	27 (36%)	
**T3**		10 (4.1%)	4 (5.3%)	
**pN Stage**				
**N0**		114 (47.1%)	51 (68%)	**0.002**^b^
**N+**		128 (52.9%)	24 (32%)	
**ALND**				
**(+)**		126 (52.1%)	25 (33.3%)	**0.005**^b^
**(-)**		116 (48%)	50 (66.7%)	
**LVI**				
**(+)**		115 (48%)	23 (31.5%)	**0.013**^b^
**(-)**		125 (52%)	50 (68.5%)	
**ER**				
**(+)**		194 (80.2%)	64 (85.3%)	0.315^b^
**(-)**		48 (19.8%)	11 (14.7%)	
**PR**				
**(+)**		167 (69%)	57 (76%)	0.245^b^
**(-)**		75 (31%)	18 (24%)	
**HER 2**				
**(+)**		52 (21.5%)	15 (20.8%)	0.90^b^
**(-)**		190 (78.5%)	57 (79.2%)	
**Molecular Subtypes**	Luminal A	63 (32%)	31 (49.2%)	**0.013**^b^
	Luminal B	134 (68%)	32 (50.8%)	
	HER-2 (+)	15 (33.3%)	6 (50%)	0.28^b^
	TNBC	30 (66.7%)	6 (50%)	
**Surgical morbidity**				
**Yes**		41 (17%)	17 (23%)	0.126^b^
**No**		201 (83%)	58 (77%)	
**5-year overall survival (95% CI)**		97% (96.98–97.02)	97% (96.96–97.04)	0.976^c^
**5-year local recurrence free survival (95% CI)**		99% (98.98–99.02)	97% (96.96–97.04)	0.377^c^
**5-year disease free survival (95% CI)**		95% (94.96–95.04)	93% (92.94–93.06)	0.361^c^

*ER* estrogen receptor, *PR* progesterone receptor, *BMI* body mass index, *IDC* invasive ductal carcinoma, *TNBC* triple negative breast cancer, *ALND* axillary lymph node dissection, *LVI* lymphovascular invasion, *ILC* invasive lobular carcinoma

^a^Mann Whitney *U* test

^b^Chi-square test

^c^Log-rank

### Surgical morbidity

The major and minor complication (increased seroma, hematoma, wound infection or dehiscence, ecchymosis, etc.) rates were 17% in PM + MLDF group and 23% in M + I group (*p* > 0.05), respectively. Secondary surgical procedures were needed in 39 (16%) patients in group 1. These were re-excision for positive surgical margins in 34 patients (14%) and re-operation due to hematoma in five patients (2.06%). Latissimus muscle flap fibrosis or necrosis developed in seven patients (2.89%) during follow-up, and it did not require re-operation. Eight (16.6%) patients in group 2 required secondary surgery; these were nipple-areola complex excision in 5 patients (6.66%) due to margin positivity or necrosis. In M + I group, prosthesis was removed in 3 patients (4%) due to infection or skin flap necrosis.

**Fig. 6:**
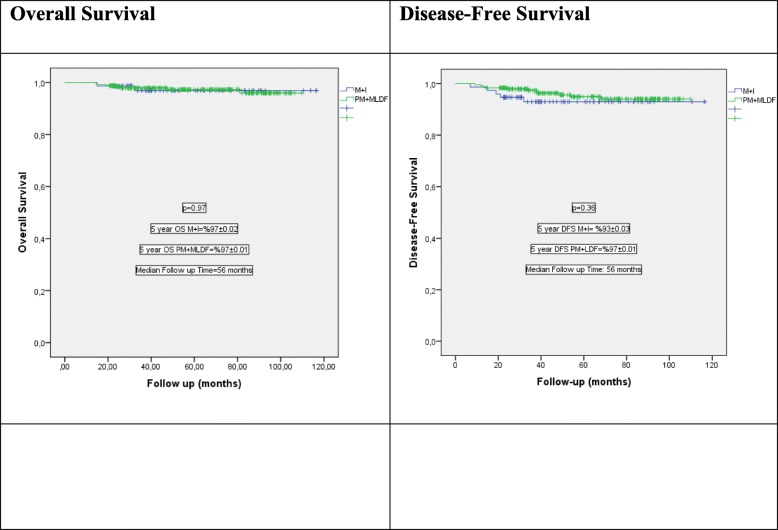
Kaplan-Meier survival plots of overall survival (OS) and disease-free survival (DFS)

Local recurrence was seen in 3 patients in group 1 and 2 patients in group 2, respectively (*p* = 0.377). The 5-year-systemic recurrence and overall survival rates were similar between the two groups (*p* > 0.05, Table 1, Fig. 6).

### Quality of life

When the quality of life was assessed by using the EORTC QLQ C30 and BR23 questionnaires, it was seen that the body image perception (*p* ≤ 0.001) and nausea/vomiting score (*p* = 0.024) were significantly better in PM + MLDF group whereas physical function score was significantly better in M + I group (*p* = 0.012, Table 2). Despite of significantly higher ALND and radiation therapy rate in group 1, there were no differences regarding arm symptoms between groups. Other parameters were similar between two groups (Table 2).

**Table 2:** Comparison of two groups by using the EORTC QLQ C30 and BR23 questionnaires

	PM + MLDF	M + I	***p*** value
**Global health status**^a^	83.3 (0–100)	83.3 (33.3–100)	0.21
**Physical function**^a^	86.6 (26.6–100)	93.3 (33.3–100)	**0.012**
**Role function**^a^	100 (0–100)	100 (50–100)	0.053
**Emotional function**^a^	83.3 (0–100)	83.3 (33–100)	0.705
**Cognitive function**^a^	100 (0–100)	100 (0–100)	0.175
**Social function**^a^	83.3 (0–100)	66.6 (17–100)	0.205
**Financial problems**^b^	33.3 (0–100)	33.3 (0–100)	0.58
**Dyspnea**^b^	0 (0–100)	0 (0–66.6)	0.483
**Pain**^b^	16.6 (0–100)	16.6 (0–100)	0.155
**Nausea/vomiting**^b^	0 (0–66.6)/3.04	0 (0–16.6)/0.29	**0.024**
**Fatigue**^b^	33.3 (0–100)	33.3 (0–88.8)	0.214
**Sleep disturbances**^b^	0 (0–100)/21.1	0 (0–100)/14.8	**0.057**
**Appetite loss**^b^	0 (0–100)	0 (0–66.6)	0.384
**Constipation**^b^	0 (0–100)	0 (0–100)	0.663
**Diarrhea**^b^	0 (0–100)	0 (0–67)	0.155
**Systemic therapy side effects**^b^	11 (7–28)	11 (7–24)	0.858
**Arm symptoms**^b^	22.2 (0–100)	22.2 (0–78)	0.511
**Breast symptoms**^b^	8.3 (0–100)	0 (0–67)	**0.055**
**Upset by hair loss**^b^	6.38 ± 15.23	4.35 ± 15.04	0.440
**Body image**^a^	75 (0–100)	58.3 (0–100)	**0.000**
**Future perspective**^a^	66.6 (0–100)	66.6 (0–100)	0.926
**Sexual function**^a^	4 (1–12)	4 (2–12)	0.165
**Sexual enjoyment**^a^	5.34 ± 14.33	3.37 ± 12.24	0.298

Mann-Whitney *U* test

^a^the higher values indicate higher level of functioning and quality of life; min: 0, max: 100

^b^the higher values indicate a greater severity of symptoms, min: 0, max: 100

### Cosmetic evaluation

When both groups were examined in terms of cosmesis with JBCS Cosmetic Evaluation Scale, there were no difference regarding bad or perfect-scaled patients in both groups whereas good cosmetic evaluation score was significantly higher in patients in group 1 (*p* = 0.001, Table 3).

**Table 3:** Comparison of groups by using Japanese Breast Cancer Society Cosmetic Evaluation Scale (JBCS)

JBCS Cosmetic Evaluation Score	Group 1 (PM + MLDF)	Group 2 (M + I)	***p*** value
**Bad**	1 (0.5%)^a^	2(3.6%)^a^	**0.01**
**Moderate**	85 (45.7%)^b^	35(62.5%)^a^	
**Good**	92 (49.5%)^b^	15(26.8%)^a^	
**Perfect**	8 (4.3%)^a^	4(7.1%)^a^	

Chi-Square test was used to compare the two groups

## Discussion

Breast conserving surgery (BCS) is the standard of choice for early-stage invasive breast cancer, with two thirds of women in developed countries pursuing this route [26]. With constant improvement in multimodality treatment options, there are substantial increases in overall and disease-free survival rates. The acceptable 10-year local recurrence rate after BCS is around 5% [26–28]. As a result of improved life expectancy and decreased local recurrence, the expectation for a better cosmetic outcome is becoming more important. Oncoplastic breast surgery (OBS) by using volume replacement or displacement has gained popularity in the last two decades [4]. Subcutaneous mastectomy with latissimus dorsi muscle flap usually requires implant and adds additional cost. It also has many other morbidities if we compare with PM + MLDF such as skin flap and nipple-areola necrosis, increase in duration of surgery, hospitalization, and recovery period to start systemic treatment. Saving nipple-areola complex together with most of the breast tissue has also positive psychologic effect on patients’ mood. The preferred method to reconstruct the breast after total mastectomy is implant placement. But, subcutaneous mastectomy and implant usually cause asymmetries and requires additional cosmetic approaches in contralateral breast for better and symmetric cosmetic outcomes. Most of patients with subcutaneous mastectomy and implants feel a foreign body instead of their own tissue for the rest of their lives. MLDF reconstruction increased BCS in selected patients by preventing more patients from mastectomy [18]. Breast-conserving surgery with PM + MLDF also saves body image with autolog graft and keeps oncological principles. In our breast center, the rate of BCS increased from 68 to 82% by using MLDF to fill the cavity in the last 10 years [18].

A high tumor/breast ratio (relatively large tumor and/or small size breast) decreases BCS rate in patients with early stage breast cancer. Neo-adjuvant chemotherapy (NAC) to reduce tumor size, mastectomy with implant or simple mastectomy are the commonly selected options in these patients [29, 30]. However, NAC has a limited effect on primary tumor and axilla especially in patients with luminal A molecular subtype [31]. Simple mastectomy causes loss of body image with psychological side effects [32]. The patients who underwent PM + MLDF in our series had a larger tumor diameter and smaller breast size as compared to the other group. Multicentric/and multifocal cancer rate was also 25.9% in this group. Since filling tumor cavity with peripheral glandular displacement is very difficult, autologous volume replacement with MLDF or subcutaneous mastectomy with implant is the surgical treatment of choice. We preferred partial mastectomy and MLDF due to its cost-effectivity and less morbidities than mastectomy and implant.

It has been reported that the ratio of multifocal/multicentric BC ranges from 4 to 50% in different series [33–37]. Addition of MRI to the work up increases multifocal/multicentric tumor rate [38]. Meta-analysis of data from nineteen studies showed that MRI detected additional disease in 16% of women leading to conversion from wide local excision to mastectomy in 11.3% of cases [33, 39]. Multicentric breast cancer has traditionally been among the indications for mastectomy; however, BCS can also be safely performed in these patients on a condition that tumors are completely excised with negative surgical margins with acceptable cosmetic outcomes [40]. In our study, 99 patients (31.2%) had multifocal/multicentric breast cancer in total, and 62 (25.9%) patients with multifocal/multicentric BC in group 1 had a chance to save the breast by using PM + MLDF surgical technique.

With the advances in BC treatment, the 10-year local recurrence rate following BCS is reduced from 10–15% down to 5% [41–43]. Factors such as surgical margin positivity, high histological grade, presence of lympho-vascular invasion, axillary nodal involvement, multifocality, and poor molecular sub-types have been associated with increased local recurrence after BCS [41–43]. In our study, patients in PM + MLDF group had tumors with higher histologic grade, higher positivity rate for LVI, and luminal A molecular subtype rate was lower. In spite of these statistically higher rate of poor prognostic factors in PM + MLDF group, no differences were seen between the groups in terms of 5-year local recurrence (2% and 3%) and overall survival rates (97% in both groups).

Prosthetic-based breast reconstruction was associated with increased major breast complications up to 49% [44–49]. It is expensive and results in delays for adjuvant systemic treatment. In a study evaluating surgical and patient-reported outcomes and quality of life in patients with autologous breast reconstruction after failed implant-based reconstruction, there was a statistically significant increase in overall outcomes (*p* < 0.001), satisfaction with appearance of breasts (*p* < 0.001), psychosocial well-being (*p* < 0.001), and physical well-being of the chest (*p* = 0.003) [45]. Complication rate for MLDF is reported much lower and generally include seroma formation, hematoma, flap necrosis, loss of flap, and wound infection [12, 47]. In our study, our complication rate was 17% in group 1 and 23% in group 2. There was no significant difference between the groups. The most frequently observed complications in group 1 and 2 were seroma, wound infection, and nipple-areola complex and/or flap necrosis, respectively. Secondary surgery rates in both groups were also similar.

The EORTC QLQ-C30 is an assessment technique that is widely used to measure the quality of life of patients who undergo surgery due to BC [19, 20]. In a study by Costa et al., the average value for the Global Health Status, which is an important parameter in the EORTC QLQ-C30, was reported as 62 [49], whereas this was identified as 50 in another study conducted by Pacaric et al. [50]. Most of the studies generally report high rates of sadness due to the loss of hair as a result of adjuvant chemotherapy [51]. In our study, the Global Health Status rates were 83.3 in both groups. These high rates in our study can be attributed to the time gap between the surgery and filling out the questionnaire. In the previous studies, the questionnaires were filled shortly after the surgery; however in our study, questionnaire was filled after all modalities of the treatment were completed. Nevertheless, we were able to achieve high quality of life rates with each surgical technique.

Different results have been reported in the literature based on the comparisons of EORTC QOL C30 and BR23 results of patients who received PM or mastectomy [19, 20, 50, 51]. Slowik et al. reported no significant difference influencing the overall quality of life as per the type of surgery performed in neither of the parameters of the questionnaire [52]. In our study, the body image parameter of patients who received PM + MLDF was found significantly higher than that of the patients who received M + I. We attribute the perception of a better body image in PM + MLDF group to the autogenous quality of the reconstruction performed, and we think that this is a very important factor. This result makes PM and autogenous reconstruction as the preferred option and should be leaned on when deciding surgical options. Another important aspect of this study was arm symptoms. Despite of significantly increased rate of ALND and the latissimus dorsi muscle used for reconstruction in Group 1, arm symptoms were similar in two groups (*p* = 0.511).

Cosmetic evaluation is crucial and must be done in every study comparing two different breast surgery techniques. The Japanese Breast Cancer Society Cosmetic Evaluation Scale has been used widely for this purpose [21]. Our study is the first study in the literature that uses this scale to compare cosmetic outcome between PM + MLDF and M + I. When we look at the results, patients underwent PM + MLDF demonstrated significantly good outcome in comparison to M + I.

Latissimus dorsi musculo-cutaneous or partial muscle flap has been used for reconstruction in patients with BC for decades [8–18]. It is also an important opportunity to use this muscle for patients with local recurrence after BCS. The most important limitation of PM + MLDF procedure is to eliminate this opportunity in these patients if they have local recurrence. In our cohort, there were only 3 local recurrences in the 5-year follow-up. However, other alternative reconstruction techniques (abdominal flaps etc.) may be used in patients with local recurrence after PM + MLDF. The limitation of our study is its retrospective design.

## Conclusion

The patients in our study who underwent PM + MLDF reconstruction had a significantly superior cosmetic outcome and a better QoL as compared to the patients who had M + I reconstruction. The two techniques do not show any differences with respect to complications, morbidity or survival rates. The PM + MLDF technique may save breasts in many patients and cost and morbidities of prosthetic implant reconstruction should be taken into consideration especially in low-middle income countries.

## Data Availability

All patients were registered in our breast center archive for 30 years and clinical, pathological, surgical, and survival characteristics of patients were retrieved from this breast cancer specific archive. If it requires, we will be happy to share our data with journal editorial board or reviewers.
